# Case Report: Pancreaticopleural fistula with an atypical tract in a child with bulging chest

**DOI:** 10.3389/fped.2023.1278463

**Published:** 2023-10-20

**Authors:** Zi Yin Shang, Chun Hong, Cui Fen Liu

**Affiliations:** Department of Pediatric Thoracic Surgery, Guangdong Women and Children Hospital, Guangzhou, China

**Keywords:** pancreaticopleural fistula, magnetic resonance cholangiopancreatography, pancreatic lesions, pleural effusion, child

## Abstract

Pancreaticopleural fistula (PPF) is a rare but serious complication caused by pancreatic lesions that presents primarily with respiratory tract symptoms and pleural effusion. We report a paediatric case of PPF without any respiratory symptoms throughout the course of the disease, including cough or shortness of breath, with only a bulging chest as the first symptom. Imaging revealed a large left pleural effusion and Magnetic Resonance Cholangiopancreatography (MRCP) revealed a fistula formed between the pancreatic tail and the pleural cavity, which penetrated the diaphragm and opened in the central tendon of the diaphragm. The patient eventually underwent resection of the pancreatic tail lesion and repair of the diaphragmatic fistula and recovered soon thereafter.

## 1. Introduction

In adults, pancreaticopleural fistula (PPF) is most often seen in patients with chronic alcohol-induced pancreatitis, with pancreatitis and pancreatic pseudocysts as the principal causes. There have been few reports of PPF in children, and most cases are of unknown cause, but may be due to viral infections, trauma, medication, and pancreas divisum. PPF presents primarily with respiratory tract symptoms and pleural effusion (1). However, abdominal symptoms are not prominent, which can easily lead to misdiagnosis or missed diagnosis. We present a case of an 8-year-old child with no respiratory symptoms during the course of the disease such as cough and shortness of breath and only a bulging chest as the first symptom. Magnetic Resonance Cholangiopancreatography (MRCP) revealed the presence of a fistula between the pancreatic tail and the thorax with a very rare tract and opening location.

## 2. Case report

**Figure 1:**
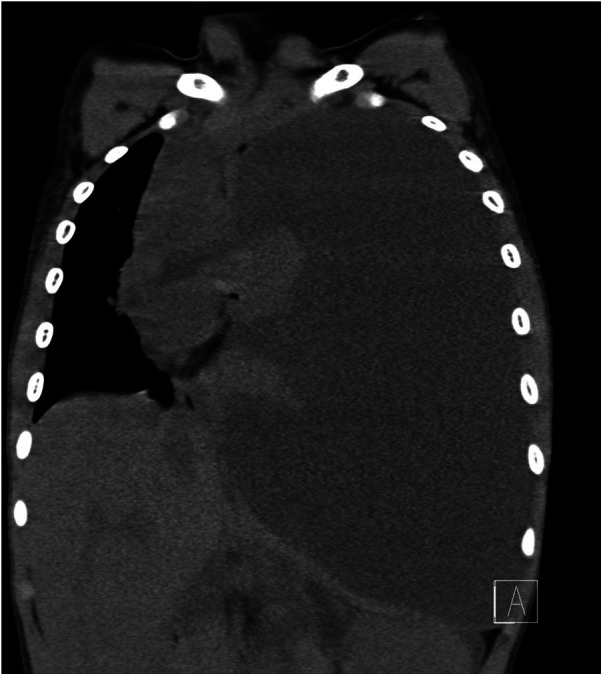
Computed tomography indicating large left pleural effusion.

An 8-year-old boy presented to the clinic with gradual bulging of the left thorax for the prior 2 weeks. The child had been treated with oral medication for recurrent vomiting over the past 2 months and had lost 5 kg of body weight over the past 2 months. Upon physical examination, the left thorax was fuller and exhibited more bulging than the right side. Left-sided respiratory movements were weakened, vocal fremitus was significantly weaker on the left side than on the right. Percussion of the left lung indicated no breath sounds in the left chest cavity, and the heart sound boundary was shifted to the right. Chest computed tomography (CT) examination revealed a large left pleural effusion with left lung atelectasis and a rightward shift of the mediastinum, as well as ascites (Figure 1); the patient was admitted to the hospital.

After admission, the child underwent emergency closed-chest drainage, and dark red pleural fluid was withdrawn. A total of 1,920 ml of fluid was drained on the first day. The results of retained pleural fluid tests were as follows: Bacterial smear and culture were negative; two tests of exfoliated cells and acid-fast bacillus were negative; metagenomic tests for pathogenic microorganisms were negative. The Xpert MTB/RIF assay was negative. Complete blood count, procalcitonin, and other indicators of infection were normal. To further exclude malignant pleural effusion, enhanced CT of the thorax and abdomen was performed. A mass-like space-occupying shadow measuring about 2.1 cm × 1.7 cm was found in the pancreatic tail. Enhanced scanning indicated progressive inhomogeneous enhancement.

**Figure 2:**
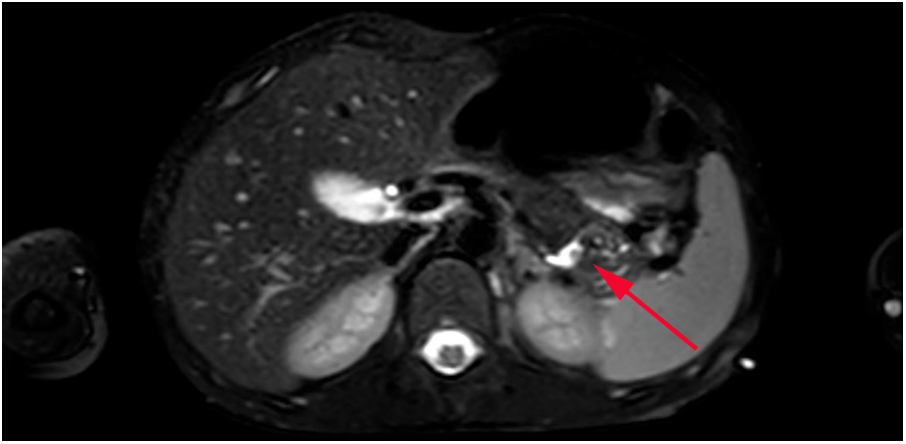
MRCP image. Arrow indicates pancreatic tail lesion.

**Figure 3:**
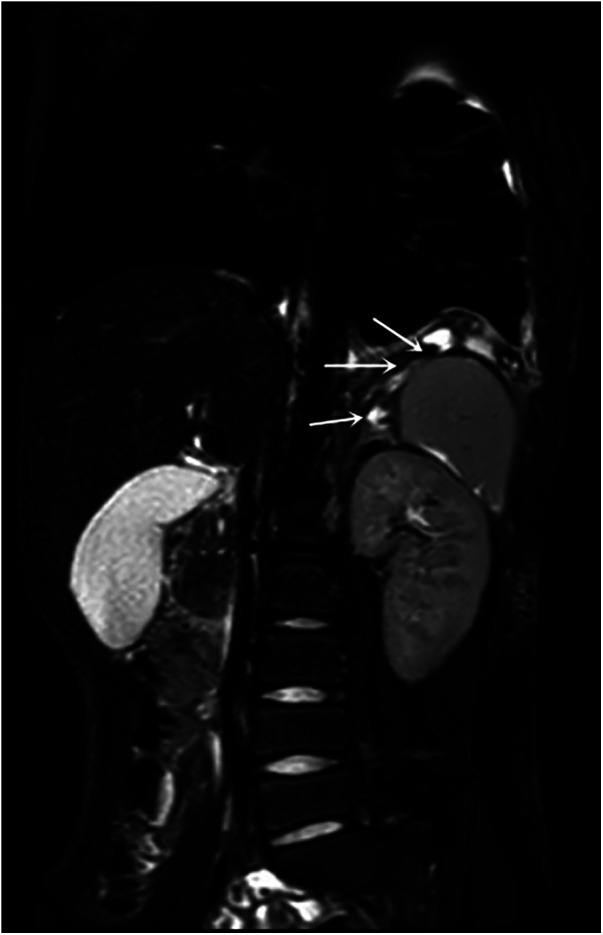
MRCP image. Arrow indicates fistula track.

The child was struck by a classmate on the right abdomen while playing at school 3 months prior. The child had an initial history of vague abdominal pain and intermittent vomiting, with symptoms gradually improving shortly thereafter. Further examination results were as follows: Pleural fluid amylase: 52,363 U/L; blood amylase: 223 U/L; and blood lipase: 639 U/L. An enhanced MRCP scan revealed that the pancreatic tail leaked pancreatic fluid, and a fistula was present between the pancreatic tail and the pleural cavity, penetrating the diaphragm via a diaphragmatic fistula 5.5 mm in width (Figures 2, 3). A diagnosis of PPF was confirmed.

The child was initially given conservative treatment, including suppression of pancreatic secretion and enteral nutrition via a jejunal nutrition tube, but the pleural effusion did not decrease significantly. After six months of postoperative surveillance, the child recovered well with recovery of appetite and weight gain, and no effusion in the thoracic or abdominal cavities were observed on CT re-examination.

## 3. Discussion

PPF is a rare but serious complication caused by pancreatic lesions. Most cases present with respiratory tract symptoms and pleural effusion as the primary manifestations. Cases with bulging chest as the first symptom are extremely rare. In this case, the pleural effusion probably increased gradually after abdominal trauma. The respiratory function of the healthy lung was able to compensate, and the child gradually developed tolerance, so there were no respiratory symptoms such as cough or shortness of breath. In addition, the child suffered from bullying at school and deliberately concealed the history of abdominal trauma out of fear. Initially, we focused on tuberculosis and cancer-related tests for haemorrhagic pleural effusion, but the diagnosis went in a different direction.

Endoscopic retrograde cholangiopancreatography (ERCP) has both diagnostic and therapeutic functions for pancreaticobiliary diseases and can be used for clear display of pancreatic damage and fistulae; its safety has been confirmed (2). However, ERCP is invasive and more technically difficult for paediatric patients, so a safer approach is needed. MRCP has the advantages of not involving radiation and being minimally invasive and can also be used for a clear display of the morphology and alignment of the pancreatic ducts and the relationship between the pancreatic ducts and fistulae with good resolution (3, 4). Elevated amylase in pleural effusion is an important foundation for the diagnosis of PPF, but acute pancreatitis, tuberculosis, oesophageal perforation, and malignant tumours of the lung or pancreas should be excluded. Some researchers believe that pleural fluid amylase above 5,000 U/L is diagnostic of PPF (3, 5, 6).

The source of fluid accumulation in PPF is thought to be due to external forces or bacterial infection, resulting in rupture of the pancreatic duct and/or pancreatic pseudocysts and leakage of pancreatic fluid. If the rupture occurs in the anterior part of the pancreas, pancreatic fluid will leak into the abdominal cavity to form ascites. If the rupture occurs in the posterior part of the pancreas, pancreatic fluid will instead leak into the retroperitoneal space, follow the path of least resistance upward, enter the mediastinum through the aorta or oesophageal fissure, and break through the pleura to form a pleural effusion (3, 5). In the present case, the pancreatic lesion was located in the posterior part of the pancreatic tail and the leaking pancreatic fluid travelled upward along the space between the medial aspect of the spleen and the adrenal gland, finally penetrating the left central tendon of the diaphragm into the thoracic cavity. The tract and opening location of the fistula were rarely reported in previous studies.

In summary, the diagnosis of PPF in the present case was based primarily on (a) recurrent haemorrhagic pleural effusion; (b) progressive weight loss; (c) amylase above 5,000 U/L in the pleural effusion; and (d) the finding of a fistula between the pancreatic tail and the diaphragm on MRCP. Treatment of PPF includes both conservative and surgical treatments. Conservative treatment includes pleural fluid drainage, parenteral nutrition, and somatostatin. Some researchers believe that patients with pancreatic duct destruction or stenosis can be cured by endoscopic pancreatic duct stent placement (7, 8), while others believe that early surgical treatment is beneficial to improve patient outcomes (9–11). We believe that restoration of anatomical continuity is more important than the reduction of pancreatic fluid secretion by conservative treatment alone.

## 4. Conclusion

PPF is very rare in children but must be considered in children with recurrent, massive haemorrhagic pleural effusion as the primary manifestation. Amylase screening should be performed. Chest and abdominal CT can assist in diagnosis, and MRCP should be used in paediatric patients to confirm the diagnosis as early as possible. For children with poor response to conservative treatment, surgical treatment should be administered as soon as possible.

## Data Availability

The original contributions presented in the study are included in the article/Supplementary Material, further inquiries can be directed to the corresponding author.
